# Supplementary material for: Impairment of Nucleolin Activity and Phosphorylation by a Trachylobane Diterpene from Psiadia punctulata in Cancer Cells
Source: Int J Mol Sci. 2022 Sep 27;23(19):11390. doi: 10.3390/ijms231911390 (PMC9570042; doi:10.3390/ijms231911390)
Supplement: Supplementary file 1 [file ijms-23-11390-s001.zip › ijms-1872402-supplementary.pdf]

Supporting Information

Table of contents

**Figure S1.**	(A) Chemical structure of compounds 1–16 and oridonin. (B) Representative western blot of the nucleolin level in the CETSA-based screening performed in Jurkat cell line	S3
**Figure S2.**	Representative western blot of the nucleolin level in CETSA experiment on the effects of compound **12** on the HeLacells at different temperatures	S3
**Figure S3.**	Representative western blot of the nucleolin level in CETSA performed using various concentrations of compound **12**	S4
**Figure S4**	SPR sensorgrams obtained for the interaction of compound **12** with hNcl (A) and RNA-BD 1,2 (B)	S4
**Figure S5**	(A) Docked pose of oridonin (salmon sticks) within RBD1,2 represented as a slate ribbon model. Only amino acids located within 4 Å of the bound ligand are displayed (white sticks) and labelled. H-bonds are depicted as dashed black lines (B) Two-dimensional ligand interaction diagram of oridonin docked pose. Positively charged amino acids are represented with dark-blue drops, negatively charged amino acids are represented with red drops, polar amino acids are represented with light-blue drops and hydrophobic amino acids are represented with green drops. H-bonds are depicted with purple arrows.	S5

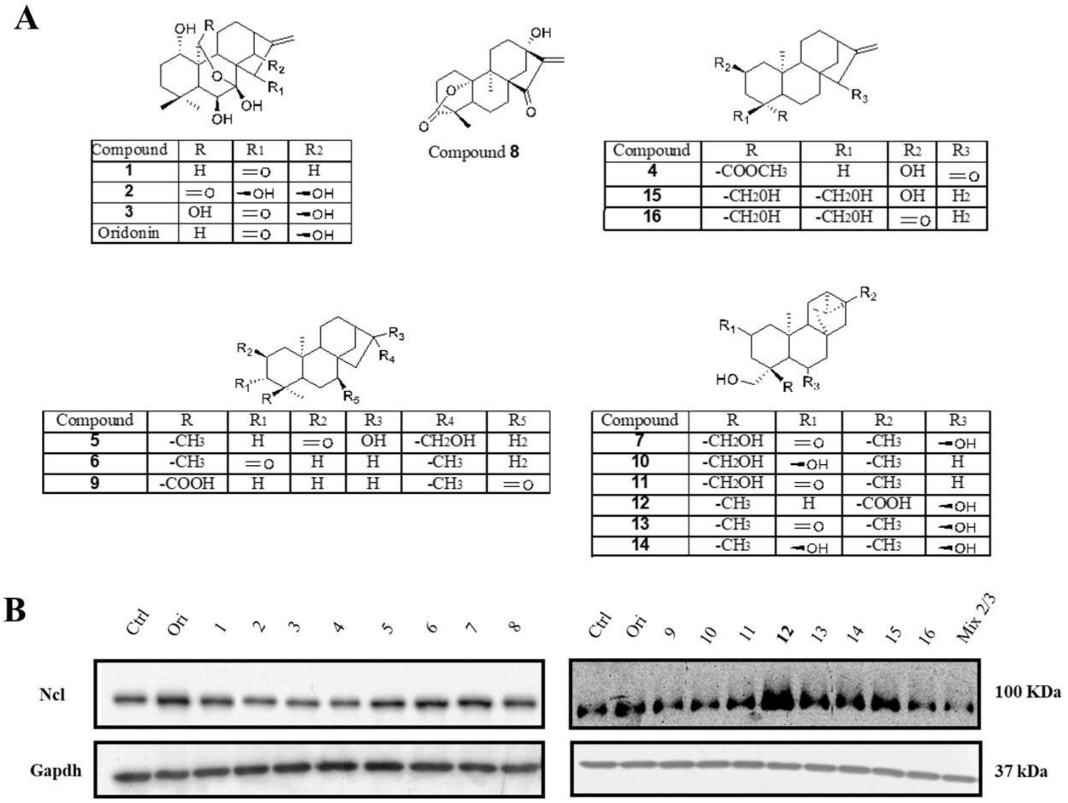

Compound	R	R_1	R_2
**1**	H	=O	H
**2**	=O	-OH	-OH
**3**	OH	=O	-OH
Oridonin	H	=O	-OH

Compound	R	R_1	R_2	R_3
**4**	$-COOCH_3$	H	OH	=O
**15**	$-CH_2OH$	$-CH_2OH$	OH	H_2
**16**	$-CH_2OH$	$-CH_2OH$	=O	H_2

Compound	R	R_1	R_2	R_3	R_4	R_5
**5**	$-CH_3$	H	=O	OH	$-CH_2OH$	H_2
**6**	$-CH_3$	=O	H	H	$-CH_3$	H_2
**9**	-COOH	H	H	H	$-CH_3$	=O

Compound	R	R_1	R_2	R_3
**7**	$-CH_2OH$	=O	$-CH_3$	-OH
**10**	$-CH_2OH$	-OH	$-CH_3$	H
**11**	$-CH_2OH$	=O	$-CH_3$	H
**12**	$-CH_3$	H	-COOH	-OH
**13**	$-CH_3$	=O	$-CH_3$	-OH
**14**	$-CH_3$	-OH	$-CH_3$	-OH

Figure S1. (A) Chemical structure of compounds **1-16** and Oridonin. (B) Representative western blot analysis of nucleolin level in the CETSA-based screening performed in Jurkat cell line

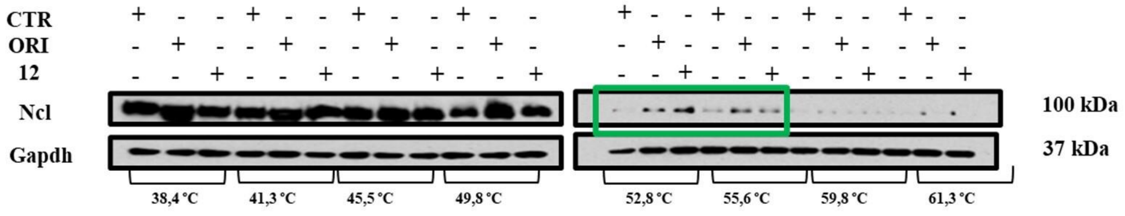

Figure S2. Representative western blot analysis of Nucleolin level in CETSA experiment of **12** on HeLa cells to different temperatures

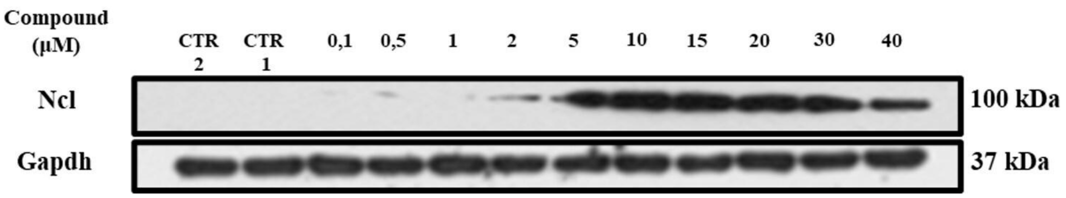

Figure S3. Representative western blot analysis of Nucleolin level in CETSA performed using various concentrations of **12**

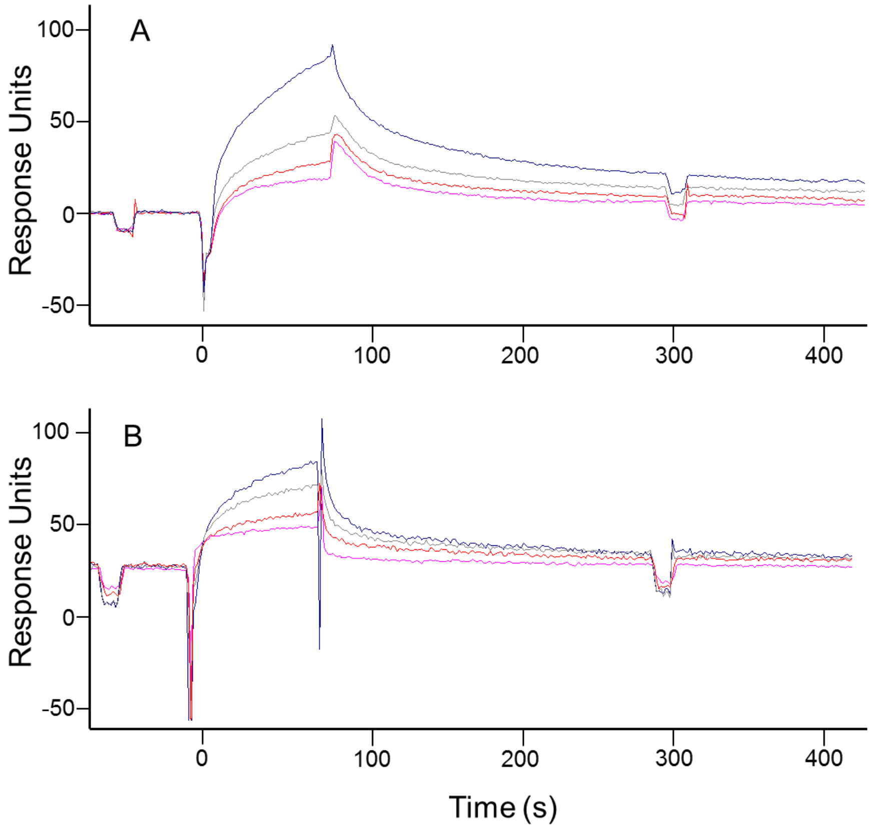

Figure S4. SPR sensorgrams obtained for the interaction of **12** with hNCL (A) and RBD 1,2 (B)

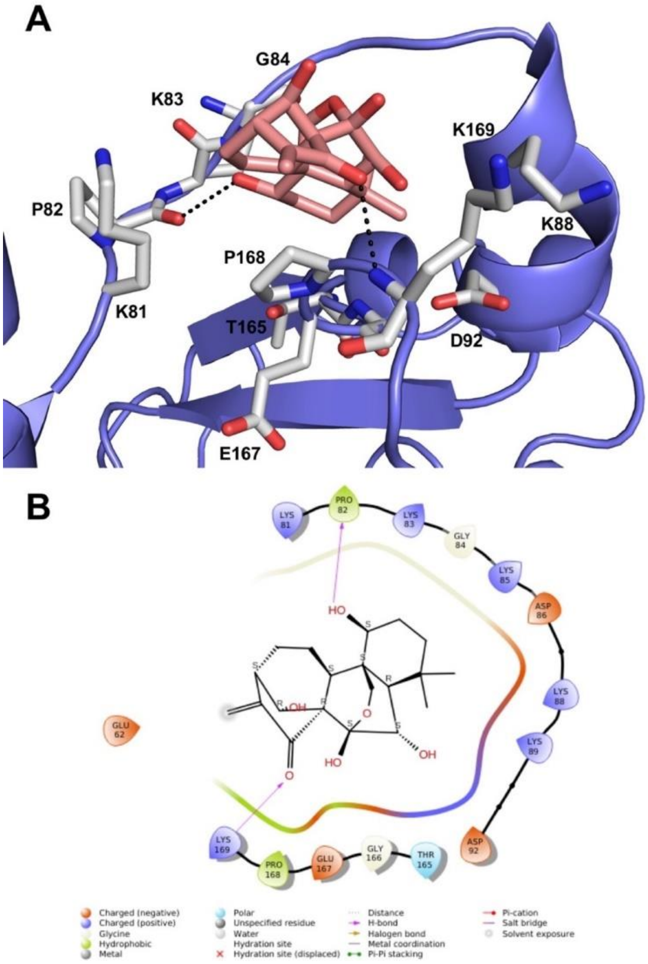

Figure S5. (A) Docked pose of oridonin (salmon sticks) within RBD1,2 represented as a slate ribbon model. Only amino acids located within 4 Å of the bound ligand are displayed (white sticks) and labelled. H-bonds are depicted as dashed black lines (B) Two-dimensional ligand interaction diagram of oridonin docked pose. Positively charged amino acids are represented with dark-blue drops, negatively charged amino acids are represented with red drops, polar amino acids are represented with light-blue drops and hydrophobic amino acids are represented with green drops. H-bonds are depicted with purple arrows.
